# ﻿*Campanophyllummicrosporum* (Agaricales, Agaricomycetes), *Caloceramultiramosa*, and *Dacrymycesnaematelioides* (Dacrymycetales, Dacrymycetes), three new species from Yunnan Province, southwestern China

**DOI:** 10.3897/mycokeys.107.125571

**Published:** 2024-08-13

**Authors:** Yuan-Hao Ma, Ping Liu, Hong-Mei Chai, Min Zeng, Yi-Yun Guo, Wei-Min Chen, Yong-Chang Zhao

**Affiliations:** 1 Biotechnology and Germplasm Resources Institute, Yunnan Academy of Agricultural Sciences, Kunming 650205, China Biotechnology and Germplasm Resources Institute, Yunnan Academy of Agricultural Sciences Kunming China; 2 Yunnan Provincial Key Lab of Agricultural Biotechnology, Kunming 650205, China Yunnan Provincial Key Lab of Agricultural Biotechnology Kunming China; 3 Key Lab of Southwestern Crop Gene Resources and Germplasm Innovation, Ministry of Agriculture and Rural Affairs, Kunming 650205, China Ministry of Agriculture and Rural Affairs Kunming China

**Keywords:** Basidiomycota, new taxon, phylogenetic analyses, taxonomy

## Abstract

Three new species belonging to Basidiomycota from southwestern China are described based on morphological and molecular data. *Campanophyllummicrosporum* is morphologically characterized by dorsally pseudostipitate, pale orange to brownish orange pileus, excentric to lateral pseudostipe, crowded lamellae, cylindrical-ellipsoid basidiospores 3.0–4.2 × 1.7–2.2 µm, narrowly clavate to clavate basidia 14.5–23.0 × 3.0–4.2 µm, and cylindrical to clavate cheilocystidia 22.0–55.0 × 5.0–10.8 µm. *Caloceramultiramosa* is morphologically characterized by stipitate, yellowish to orange, dendroid, and dichotomously branched basidiomata, cylindrical to clavate basidia 36.5–52.5 × 3.8–6.1 µm, navicular or reniform, 1–5-septate mature basidiospores 10.4–16.7 × 5.2–7.4 µm. *Dacrymycesnaematelioides* is morphologically characterized by stipitate and cerebriform, orange to light brown basidiomata, cylindrical to clavate, smooth or roughened basidia 38.5–79.5 × 6.5–10.6 µm, broadly and elliptic-fusiform, 7-septate mature basidiospores 18.5–28.6 × 8.9–13.8 µm. These three new species are supported by the phylogenetic analyses using maximum likelihood (ML) and Bayesian inference (BI) analyses with combined nuclear ribosomal DNA (rDNA) internal transcribed spacer (ITS) and large ribosomal subunit (LSU) sequences. Full descriptions and photographs of these new species are provided.

## ﻿Introduction

The monotypic genus *Campanophyllum* Cifuentes & R.H. Petersen was proposed to accommodate *Lentinusproboscideus* Fr., traditionally contains dorsally pseudostipitate pileus with tricholomataceus, excentric to lateral pseudostipe, crowded lamellae, cylindrical-ellipsoid spores, cylindrical, clavate to utriform cheilocystidia, and grows on rotten wood (Cifuentes et al. 2003). *L.proboscideus* was combined into the genus *Campanophyllum* in the family Cyphellaceae, and designated a neotype by Cifuentes et al. (2003). In addition, the authors demonstrated that this species represents a novel species of a novel genus distinct from its closest relatives through a comprehensive analysis of morphological characteristics, molecular data, and sexual compatibility (Cifuentes et al. 2003). The species was found in montane forests in Colombia, Costa Rica, Ecuador, Mexico, and Panama, and is currently being assessed for inclusion in the IUCN red list as endangered (https://redlist.info/iucn/species_view/488791/) (Cifuentes et al. 2003; Reschke et al. 2021). Recently, researchers also collected the species of *C.proboscideum* in India and studied its fungal extracts, which are rich in natural antioxidants and highly effective antimicrobial activity (Borthakur et al. 2020). However, the specimens collected from India may not be of *C.proboscideum*, but of another species, due to the huge differences in the ITS sequences. The absence of detailed morphological descriptions precludes the ability to ascertain the specific species to which the specimen belongs.

*Calocera* (Fr.) Fr. and *Dacrymyces* Nees are the two major polyphyletic groups in the family Dacrymycetaceae, characterized by pulvinate to dendroid or cerebriform basidiomata (Shirouzu et al. 2007, 2009, 2013, 2017; Zamora and Ekman 2020; Fan et al. 2021; Lian et al 2022; Zamora et al. 2022). Their classifications in Dacrymycetes are based on morphology and have remained unaltered (McNabb 1965, 1973; Shirouzu et al. 2017; Zamora and Ekman 2020). However, this morphology-based classification has often conflicted with the results of molecular phylogenetic analyses, and *Calocera*, *Dacrymyces*, and *Dacryopinax* G.W. Martin have been shown to be non-monophyletic genera (Shirouzu et al. 2013, 2017; Zamora and Ekman 2020). Phylogenetic analysis shows that species of *Calocera* and *Dacrymyces* are distributed in many clades of the family Dacrymycetaceae. The three species of *C.cornea* (Batsch) Fr., *C.lutea* (Massee) McNabb, and *C.fusca* Lloyd were clustered into three distinct clades, rather than forming a single clade, despite belonging to the same genus, and many of the species of *Dacrymyces* were grouped with other genera in one clade (Shirouzu et al. 2017; Zamora and Ekman 2020).

*Calocera* is ecologically saprobic, causing brown rot except *C.viscosa* (Pers.) Bory, and *C.lutea* which are white rot species (Shirouzu et al. 2009, 2013). According to the Index Fungorum (https://www.indexfungorum.org) as of June 2024, 95 species names of *Calocera* are recorded. In China, only six species have been reported: *C.sinensis* McNabb, *C.hunanensis* B. Liu & K. Tao, *C.mangshanensis* B. Liu & L. Fan, *C.morchelloides* B. Liu & L. Fan, *C.bambusicola* Sheng H. Wu, and *C.tibetica* F. Wu, L.F. Fan & Y.C. Dai (McNabb 1965; Liu et al. 1988; Liu and Fan 1989, 1990; Fan et al. 2021). *Dacrymyces* described by Nees (1816) based on *D.stillatus*, is treated as a genus of saprotrophic fungi (Shirouzu et al. 2009; Zamora and Ekman 2020; Zamora et al. 2022). A total of 234 species names of *Dacrymyces* is recorded in the Index Fungorum in June 2024, and the genus appears to be the most polyphyletic in the phylogeny of the Dacrymycetales (Shirouzu et al. 2017; Zamora and Ekman 2020; Savchenko et al. 2021; Zamora et al. 2022).

The Laojun Mountain is one of the main parts of the Three Parallel Rivers of Yunnan Protected Areas (TPRYPA), the World Natural Heritage Site, in northwest Yunnan Province, southwestern China. The TPRYPA is part of the Mountains of Southwest China Biodiversity Hotspot, which includes 12,000 plant species, 29 percent of which are found nowhere else (Zhang et al. 2010; Mittermeier et al. 2011). The Laojun Mountain is located between 26°2.80'–27°36.60'N, 99°1.20'–99°54.60'E and includes four counties, including Yulong, Jianchuan, Lanping, and Weixi, with an area of about 108,500 hm^2^ and elevations ranging from 2,100 to 4,513 m (Zhang et al. 2010). The dominant tree species in Laojun Mountain are *Abies* sp., *Acer* sp., *Betula* sp., *Cyclobalanopsis* sp., *Fargesia* sp., *Lithocarpus* sp., *Picea* sp., *Pinus* sp., *Quercus* sp., *Rhododendron* sp., and *Sorbus* sp. (Wu and Zhu 1987).

During the investigation of the diversity of macrofungi in the Laojun Mountain, a multitude of specimens, including a dozen belonging to the *Campanophyllum* genus and several belonging to the genera *Calocera* and *Dacrymyces*, were collected from July to September 2019–2023. In this study, the specimens of these three new species were collected from the same position in a deciduous forest of the Laojun Mountain. With the combination of morphological observations and phylogenetic analyses, we described three new species, namely *Campanophyllummicrosporum*, *Caloceramultiramosa*, and *Dacrymycesnaematelioides*.

## ﻿Materials and methods

### ﻿Specimen collection, morphological observation, and isolation

The fungal specimens used in this study were collected from the Laojun Mountain in northwestern Yunnan Province, China. After collection, the specimens were dried in an electric drier at ca. 45 °C, and deposited in the Herbarium of Cryptogams, Kunming Institute of Botany of the Chinese Academy of Sciences (HKAS). Macromorphological characteristics and habitats were obtained from field notes and photographs. Color codes were based on Kornerup and Wanscher (1978). Micromorphological features were observed from the dried specimens and measured and photographed in 5% KOH solution (w/v) and 1% Congo Red solution (w/v) using a Leica DM6 B upright light microscope and Leica Application Suite X (LAS X, version 3.7.5). In the description of basidiospores, the abbreviations m/n/p denote m basidiospores measured from n basidiomata of p collections. The dimensions of the microscopic structures are given as (a–) b–c (–d), in which b–c contains at least 90% of the measured values, and (a–) and (–d) are the extreme values provided in parentheses. The Q value stands for the ratio of length/width of an individual basidiospore and basidium, and L_m_/W_m_/Q_m_ refers to the average length/width/Q value of all basidiospores (Na et al. 2022; Wei et al. 2024). The strains of *Campanophyllummicrosporum* were isolated from the inner tissue of fresh basidiomata using a Yeast Extract Peptone Dextrose Agar (YPD) Medium consisting of 2 g yeast extract (Beijing Aoboxing Biotech Co., Ltd.), 2 g peptone (Beijing Aoboxing Biotech Co., Ltd.), 20 g dextrose (Tianjin Fengchuan Chemical Reagent Co., Ltd.), 13 g agar (Biosharp Life Sciences), and 1000 mL distilled water. The living cultures were preserved at the National Germplasm Bank of Edible Mushroom (Yunnan). Their isolate IDs are YAASM 7490 and 7491.

### ﻿DNA extraction, PCR amplification and sequencing

The genomic DNA was extracted from the dry specimens and cultured mycelia using the Fungal gDNA kit GD2416 (Biomiga CA, USA) following the manufacturer’s instructions. The entire ITS and partial LSU of the nrDNA region were amplified from the total DNA using the primer pair ITS5/ITS4 (White et al. 1990) and LR0R/LR7 (Vilgalys and Hester 1990; Moncalvo et al. 2000), respectively, and no DNA template was used as the negative control. The PCR cycling for the amplification of both ITS and LSU was as set follows: an initial denaturation at 95 °C for 3 min, followed by 34 cycles of 95 °C for 30 s, 56 °C for 1 min, 72 °C for 1 min, and a final extension at 72 °C for 5 min. The PCR products were sequenced bi-directionally by Tsingke Biotechnology Co., Ltd. Kunming, China. Newly generated sequences of both directions were assembled using the software SeqMan version 11.1.0 (DNASTAR, Inc.), and submitted to GenBank (accession nos. ITS: PP550870–PP550882, LSU: PP550017–PP550027).

### ﻿Sequence alignment and phylogenetic analyses

The sequences used in this study were those retrieved from GenBank combined with newly generated sequences. Taxon information and GenBank accession numbers of all the sequences are listed in Table 1. All sequences were aligned using the software MAFFT 7.503 (Katoh and Standley 2013) with the default settings and edited manually using BioEdit 7.2.5 (Hall 1999). After alignment, the ITS and LSU datasets were concatenated using the program SequenceMatrix 1.8.1 (Vaidya et al. 2011). Phylogenetic analyses of Cyphellaceae and Dacrymycetaceae were performed using maximum likelihood (ML) and Bayesian inference (BI) analyses based on the sequences matrix on the personal computer. The best-fit models for the concatenated ITS+LSU dataset were selected according to the Akaike Information Criterion (AIC) in jModelTest 2.1.10 (Guindon and Gascuel 2003; Darriba et al. 2012). ML analyses of the concatenated ITS+LSU dataset of Cyphellaceae and Dacrymycetaceae were performed using RAxML-NG 1.1.0 (Kozlov et al. 2019) under the GTR+I+G model with 1,000 bootstrap replicates. BI analyses of Cyphellaceae and Dacrymycetaceae were implemented using MrBayes 3.2.7 (Ronquist et al. 2012) under the GTR+I+G model. There were four independent runs, each of which had four chains for 15,000,000 generations sampling from the posterior distribution every 1000^th^ generation. The first 25% of the sampled trees were discarded as burn-in, while the remaining trees were used to obtain the Bayesian posterior probabilities of the clades. The constructed phylogenetic trees were visualized and edited in FigTree 1.4.4 (http://tree.bio.ed.ac.uk/software/figtree/) and Adobe Illustrator 25.3.1. *Flammulinavelutipes* (Curtis) Singer, was used as an outgroup in the phylogeny of Cyphellaceae (Vizzini et al. 2022), while *Suilluspictus* (Peck) Kuntze, and *Coprinuscomatus* (O.F. Müll.) Pers., were used as the outgroup in the phylogeny of Dacrymycetaceae (Shirouzu et al. 2013). The final alignments and the retrieved topologies were deposited in TreeBASE (http://purl.org/phylo/treebase/phylows/study/TB2:S31416) with submission ID 31416.

**Table 1.** Taxa used in the phylogenetic analyses and their corresponding GenBank accession numbers. Newly generated sequences are in bold. Type materials are marked with ‘T’.

Species	Isolate ID /Voucher	Country	GenBank Accession Numbers		Reference
			ITS	LSU	
Agaricomycetes					
***Campanophyllummicrosporum***	– /**HKAS 133167**	**China**	**PP550870**	**PP550018**	**this study**
***C.microsporum***	– /**HKAS 133168**	**China**	**PP550871**	**PP550019**	**this study**
***C.microsporum***	– /**HKAS 133169**	**China**	**PP550872**	**PP550020**	**this study**
***C.microsporum***	– /**HKAS 133170^T^**	**China**	**PP550873**	**PP550017**	**this study**
*C.proboscideum*	– /TENN56402	Mexico	AY230866	AY230866	Cifuentes et al. (2003)
*C.proboscideum*	– /TENN56427	Mexico	AY230867	AY230867	Cifuentes et al. (2003)
*C.proboscideum*	– /PA46	Panama	MW386067	–	Reschke et al. (2021)
*C.proboscideum*	– /PAN327	Panama	MW386071	–	Reschke et al. (2021)
*C.proboscideum*	– /PAN373	Panama	MW386072	–	Reschke et al. (2021)
*C.proboscideum*	– /NEHU.MBSRJ. 38	India	KP843881	–	unpublished
*Chondrostereumcoprosmae*	– /PDD: 119544	New Zealand	OL709440	–	unpublished
*C.coprosmae*	– /PDD: 89940	New Zealand	OL709437	OL709436	unpublished
*C.purpureum*	HHB-13334-sp. /–	USA	AF518607	–	Hibbett and Binder (2002)
*C.purpureum*	SFI-B18 /–	Ireland	MT535785	MT559785	unpublished
*C.purpureum*	14-2300 /–	USA	MG774405	–	Merlet et al. (2018)
*C.purpureum*	CBS 350.53 /–	France	MH857241	MH868775	Vu et al. (2019)
*C.vesiculosum*	– /PDD: 119640	New Zealand	OR607672	–	unpublished
*Cunninghammycesumbonatus*	– /He 5316	China	MW557955	–	unpublished
*C.umbonatus*	– /He 5311	China	MW557940	MW557954	unpublished
*C.umbonatus*	– /He 5313	China	MW557941	–	unpublished
*Cyphelladigitalis*	– /PVKU3421	Czech Republic	OM837174	–	Holec et al. (2022)
*C.digitalis*	Thorn-617 /–	USA	AY293175	–	Binder et al. (2005)
*C.digitalis*	CBS 679.82 /–	USA	DQ486698	AY635771	Matheny et al. (2006)
*Gloeostereumincarnatum*	G1905 /HCC-3	Russia	MK278092	–	Varga et al. (2019)
*G.incarnatum*	– /KUC20131022-28	South Korea	KJ668540	KJ668393	Jang et al. (2015)
*G.incarnatum*	3332 /–	Sweden	AF141637	–	Parmasto and Hallenberg (2000)
*G.incarnatum*	– /NIFoS 1948	South Korea	MH992519	–	unpublished
*G.incarnatum*	BCC 41461 /–	Thailand	KY614001	KY614002	unpublished
*G.cimri*	CBS 145006^T^ /–	Netherlands	MT023735	MN266884	Ahmed et al. (2020)
*Granulobasidiumvellereum*	G0482 /DK 2781	Poland	MK278094	–	Varga et al. (2019)
*G.vellereum*	CBS 52.84 /–	USA	AY745729	–	unpublished
*G.vellereum*	– /B. Gilsenius (GB)	Sweden	DQ677490	–	Larsson (2007)
*G.vellereum*	BAFCcult 4367 /–	Argentina	KC881193	–	Robles et al. (2015)
*G.vellereum*	TJU_NOV19 /–	China	OM237077	–	unpublished
*Incrustocalyptellacolumbiana*	– /K:237992	United Kingdom	MW830122	–	unpublished
*Flammulinavelutipes*	AFTOL-ID 558 /–	USA	AY854073	AY639883	unpublished
Dacrymycetes					
*Caloceracornea*	CBS 124.84 /–	Canada	AB712437	AB472738	Shirouzu et al. (2013)
*C.cornea*	ICMP 20465 /PDD 104991	New Zealand	LC131403	LC131362	Shirouzu et al. (2017)
*C.cornea*	AFTOL-ID 438 /–	unknown	AY789083	AY701526	unpublished
*C.cornea*	ICMP 21223 /PDD 107847	New Zealand	LC131404	LC131363	Shirouzu et al. (2017)
*C.cornea*	– /UPS F-940774	Sweden	MN595626	MN595626	Zamora and Ekman (2020)
*C.cornea*	– /CWU(MYC)6922	Ukraine	MW191969	MW159089	Savchenko et al. (2021)
*C.furcata*	– /H:Spirin 10949	Russia	MW191975	MW159088	Savchenko et al. (2021)
*C.furcata*	– /TU135016	Estonia	MW191958	MW159087	Savchenko et al. (2021)
*C.tibetica*	– /Dai20171^T^	China	MW549777	MW750403	Fan et al. (2021)
*C.tibetica*	– /Dai20178	China	MW549778	MW750404	Fan et al. (2021)
***C.multiramosa***	– /**HKAS 133171^T^**	**China**	**PP550874**	**PP550021**	**this study**
***C.multiramosa***	– /**HKAS 133172**	**China**	**PP550875**	**PP550022**	**this study**
***C.multiramosa***	– /**HKAS 133173**	**China**	**PP550876**	**PP550023**	**this study**
*C.viscosa*	AFTOL-ID 1679 /MW 591	Germany	DQ520102	DQ520102	unpublished
*C.viscosa*	TUFC12873 /TNS-F-15704	Japan	AB712439	AB299048	Shirouzu et al. (2013)
*C.viscosa*	– /UPS F-940773	Sweden	MN595628	MN595628	Zamora and Ekman (2020)
*C.viscosa*	– /CWU(MYC)6937	Ukraine	MW191970	MW159090	Savchenko et al. (2021)
*Cerinomycesaculeatus*	– /TUMH61942 (TUFC50098)^T^	Japan	MW191955	MW159053	Savchenko et al. (2021)
*C.atrans*	TUFC 30545 /–	Canada	AB712443	AB712423	Shirouzu et al. (2013)
*C.borealis*	– /O160848^T^	Norway	MW191890	MW159042	Savchenko et al. (2021)
*C.brevisetus*	– /URM:Chikowski 1544^T^	Brazil	MW191886	MW159046	Savchenko et al. (2021)
*C.creber*	– /UPS:F-946512^T^	Spain	MW191985	MW191985	Savchenko et al. (2021)
*C.enatus*	TUFC12876 /TNS-F-21034	Japan	AB712441	AB472696	Shirouzu et al. (2013)
*C.ramosissimus*	CFMR:FP-150848^T^ /–	Belize	AB712446	AB712426	Shirouzu et al. (2013)
*Dacrymycesburdsallii*	CFMR:HHB-6908^T^ /–	USA	AB712444	AB712424	Shirouzu et al. (2013)
*D.capitatus*	– /Dai 20023	China	OL587808	OL546776	unpublished
*D.capitatus*	CBS 293.82 /–	Canada	AB712450	AB472741	Shirouzu et al. (2013)
*D.ceraceus*	CFMR:HHB-8969^T^ /–	USA	AB712442	AB712422	Shirouzu et al. (2013)
*D.chrysocomus*	– /UPS:F-940136	Spain	MN595629	MN595629	Zamora and Ekman (2020)
*D.chrysocomus*	– /UPS:F-940134	Sweden	MN595630	MN595630	Zamora and Ekman (2020)
*D.chrysospermus*	TUFC13115 /TNS-F-15712	Japan	AB712452	AB299073	Shirouzu et al. (2013)
*D.chrysospermus*	– /H:Spirin 10795	Russia	MW191974	MW159078	Savchenko et al. (2021)
*D.chrysospermus*	– /H:Miettinen 14818	USA	MW191961	MW159077	Savchenko et al. (2021)
*D.* aff. *Chrysospermus*	– /UPS:F-593536	Japan	MN595631	MN595631	Zamora and Ekman (2020)
*D.dictyosporus*	CFMR:HHB-8618 /–	USA	AB712454	AB712429	Shirouzu et al. (2013)
*D.estonicus*	– /UPS:F-940137	Sweden	MN595632	MN595632	Zamora and Ekman (2020)
*D.estonicus*	– /UPS:F-940138	Sweden	MN595633	MN595633	Zamora and Ekman (2020)
*D.fennicus*	– /H:Miettinen 21174	Finland	MW191957	MW159071	Savchenko et al. (2021)
*D.fennicus*	– /UPS:F-946596	Sweden	MZ147627	MZ147627	Savchenko et al. (2021)
*D.grandinioides*	– /H7008841	Kenya	MW191950	MW159076	Savchenko et al. (2021)
*D.lacrymalis*	TUFC13327 /TNS-F-15719	Japan	AB712456	AB299069	Shirouzu et al. (2013)
D.cf.minor	– /H:Miettinen 19137	Finland	MW191967	MW159080	Savchenko et al. (2021)
D.cf.minor	– /H:Miettinen 20591	Finland	MW191965	MW159079	Savchenko et al. (2021)
***D.naematelioides***	– /**HKAS 133174a^T^**	**China**	**PP550877**	**PP550024**	**this study**
***D.naematelioides***	– /**HKAS 133174b^T^**	**China**	**PP550878**	**PP550025**	**this study**
***D.naematelioides***	– /**HKAS 133174c^T^**	**China**	**PP550879**	**PP550026**	**this study**
***D.naematelioides***	– /**HKAS 133174d^T^**	**China**	**PP550880**	**PP550027**	**this study**
***D.naematelioides***	**YAASM 7490** /–	**China**	**PP550881**	–	**this study**
***D.naematelioides***	**YAASM 7491** /–	**China**	**PP550882**	–	**this study**
*D.ovisporus*	– /H:Miettinen 20787	Finland	MW191964	MW159074	Savchenko et al. (2021)
*D.ovisporus*	– /H:Spirin 11145	Norway	MW191960	MW159073	Savchenko et al. (2021)
*D.pinacearum*	– /UPS:F-593533	Japan	MN595637	MN595637	Zamora and Ekman (2020)
*D.pinacearum*	– /UPS:F-593535	Japan	MN595638	MN595638	Zamora and Ekman (2020)
*D.puniceus*	TUFC12833 /TNS-F-15711	Japan	AB712449	AB299057	Shirouzu et al. (2013)
*D.puniceus*	– /Wu180	China	OL587812	OL546780	unpublished
*D.sinostenosporus*	– /Dai 20003^T^	China	MW540888	MW540890	Lian et al. (2022)
*D.sinostenosporus*	– /Dai 20008	China	MW540889	MW540891	Lian et al. (2022)
*D.sobrius*	CFMR:RLG-13487^T^ /–	USA	AB712445	AB712425	Shirouzu et al. (2013)
*D.stenosporus*	ICMP 20488 /PDD 105018^T^	New Zealand	LC131433	LC131396	Shirouzu et al. (2017)
*D.stenosporus*	ICMP 21237 /PDD 107970	New Zealand	LC131434	LC131397	Shirouzu et al. (2017)
*D.stillatus* (*anamorph*)	– /UPS:F-939814	Sweden	MN595676	MN595676	Zamora and Ekman (2020)
*D.stillatus* (*anamorph*)	– /UPS:F-939816	Sweden	–	MN593494	Savchenko et al. (2021)
*D.stillatus* (*teleomorph*)	– /UPS:F-939814	Sweden	MN595677	MN595677	Zamora and Ekman (2020)
*D.stillatus* (*teleomorph*)	– /UPS:F-939816	Sweden	–	MN593495	Savchenko et al. (2021)
*D.subalpinus*	TUFC12834 /TNS-F-15730	Japan	AB712465	AB299060	Shirouzu et al. (2013)
*D.venustus*	– /O:Adane 150^T^	Ethiopia	MW191949	MW159075	Savchenko et al. (2021)
*Dacryonaemamacnabbii*	– /UPS:F-940949	Sweden	MN595650	MN595650	Zamora and Ekman (2020)
*D.macnabbii*	– /UPS:F-940992	Sweden	MN595653	MN595653	Zamora and Ekman (2020)
*D.macrosporum*	– /UPS:F-940998	Finland	MN595660	MN595660	Zamora and Ekman (2020)
*D.macrosporum*	– /UPS:F-941001	Finland	MN595661	MN595661	Zamora and Ekman (2020)
*D.rufum*	– /UPS:F-941005	Sweden	MN595646	MN595646	Zamora and Ekman (2020)
*D.rufum*	– /UPS:F-941012	Finland	MN595649	MN595649	Zamora and Ekman (2020)
*Dacryopinaxelegans*	– /TENN 066927	USA	MN595640	MN595640	Zamora and Ekman (2020)
*Dacryopinax* sp.	– /H7008759	Kenya	MW191959	MW159091	Savchenko et al. (2021)
*D.spathularia*	TUFC12846 /TNS-F-21048	Japan	AB712473	AB472710	Shirouzu et al. (2013)
*D.spathularia*	FCME 27539 /–	Mexico	MN733711	MN733722	Castro-Santiuste et al. (2020)
*D.spathularia*	– /H:Miettinen 20559	Indonesia	MW191976	MW159092	Savchenko et al. (2021)
*Dendrodacrysciprense*	– /UPS:F-946590^T^	Cyprus	OM519385	OM519385	Zamora et al. (2022)
*D.ciprense*	– /UPS:F-946591	Cyprus	OM519386	OM519386	Zamora et al. (2022)
*D.concrescens*	– /UPS:F-946602^T^	Sweden	OM519390	OM519390	Zamora et al. (2022)
*D.ellipsosporum*	– /UPS:F-946604^T^	Spain	OM519392	OM519392	Zamora et al. (2022)
*D.oblongisporum*	– /UPS:F-979568^T^	Spain	OM519400	OM519400	Zamora et al. (2022)
*Ditiolapeziziformis*	– /H:Haikonen 24269	Finland	MW191972	MW159070	Savchenko et al. (2021)
*D.peziziformis*	– /H:Haikonen 30097	Finland	MN595642	MN595642	Zamora and Ekman (2020)
*D.radicata*	– /H:Miettinen 20590.2	Finland	MW191966	MW159083	Savchenko et al. (2021)
*D.radicata*	– /UPS:F-939957	Sweden	MN595641	MN595641	Zamora and Ekman (2020)
*Guepiniopsisbuccina*	– /CWU(MYC)7014	Ukraine	MW191971	MW159086	Savchenko et al. (2021)
*G.buccina*	– /UPS:F-940947	Spain	MN595643	MN595643	Zamora and Ekman (2020)
*Unilacrymaunispora*	– /UPS:F-941279	Sweden	MN595667	MN595667	Zamora and Ekman (2020)
*U.bispora*	– /UPS:F-941254	Sweden	MN595670	MN595670	Zamora and Ekman (2020)
*U.bispora*	– /UPS:F-941266	Sweden	MN595674	MN595674	Zamora and Ekman (2020)
Dacrymycetes sp.	NBRC 110592 /–	Japan	LC004003	LC003884	Shirouzu et al. (2016)
*Coprinuscomatus*	AFTOL-ID 626 /–	USA	AY854066	AY635772	Shirouzu et al. (2013)
*Suilluspictus*	AFTOL-ID 717 /–	USA	AY854069	AY684154	Shirouzu et al. (2013)

## ﻿Results

### ﻿Phylogenetic analyses

**Figure 1.**
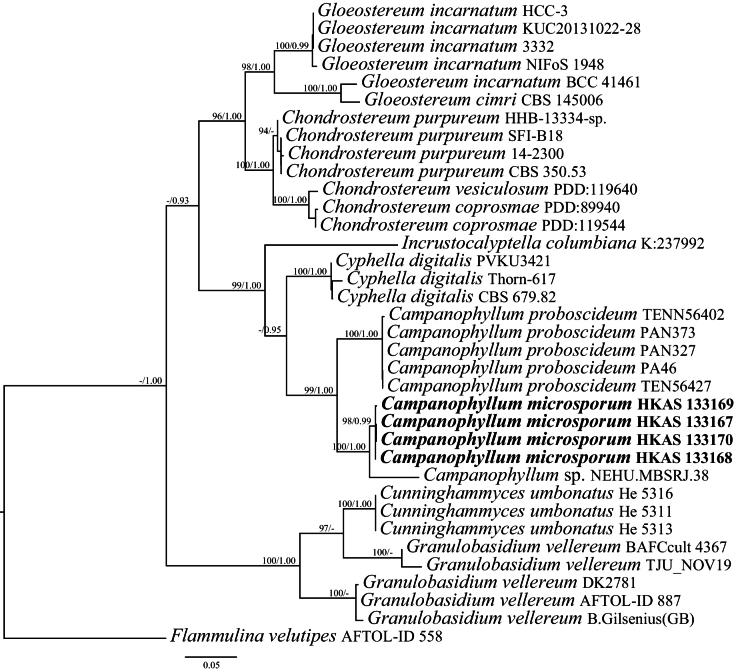
Maximum likelihood (ML) tree of Cyphellaceae based on the combined ITS+LSU dataset. ML bootstrap values (BS > 75%) and Bayesian posterior probabilities (PP > 0.95) are shown at the nodes in the order of BS/PP. The tree is rooted with *Flammulinavelutipes*. The new taxon is indicated in bold.

In the phylogeny of Cyphellaceae, 36 sequences were used for phylogenetic analyses, of which four sequences were newly generated in this study. The concatenated dataset of ITS and LSU sequences comprised a total of 1695 characters. ML and BI analyses generated similar topologies, so only the ML tree is presented along with the support values from the Maximum likelihood bootstrap (BS, >75%) values and Bayesian inference (BI) posterior probabilities (PP, >0.95) (Fig. 1). The phylogeny revealed that Cyphellaceae was divided into three clades, and these three genera of *Incrustocalyptella* Agerer, *Cyphella* Fr., and *Campanophyllum* constituted one of the three major clades with strong statistical supports (99% BS, 1.00 PP). In the ML tree, the phylogenetic results demonstrated that *Campanophyllummicrosporum* formed a distinct lineage closely related to *C.proboscideum* and *Campanophyllum* sp. (Voucher NEHU.MBSRJ. 38) with strong statistical supports (98% BS, 0.99 PP). The ITS sequences from *C.microsporum* and *C.proboscideum* were markedly different, with ca. 105 different nucleobases, and the ITS sequences of *C.microsporum* and *Campanophyllum* sp. (Voucher NEHU.MBSRJ. 38) were different with about 20 different nucleobases.

**Figure 2.**
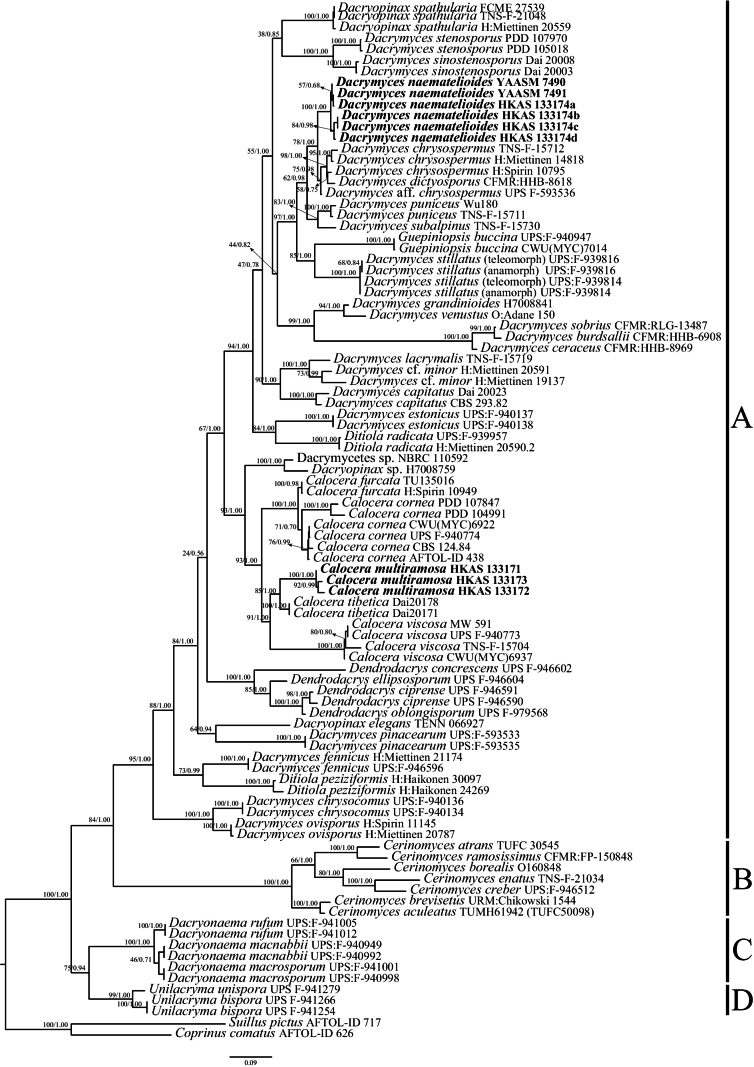
Maximum likelihood (ML) tree of Dacrymycetaceae based on the combined ITS+LSU dataset. ML bootstrap values and Bayesian posterior probabilities are shown at the nodes in the order of BS/PP. The tree is rooted with *Suilluspictus* and *Coprinuscomatus*. The new taxon is indicated in bold.

In the phylogeny of Dacrymycetaceae, the concatenated dataset of LSU and ITS sequences comprised a total of 1649 characters. 94 sequences were used for phylogenetic analyses, of which nine sequences were newly generated in this study. ML and BI analyses generated similar topologies, so only the ML tree is presented (Fig. 2). Within Dacrymycetes, we distinguish four main groups, Dacrymycetaceae (clade A), Cerinomycetaceae (clade B), Dacryonaemataceae (clade C), and Unilacrymaceae (clade D). The clade A included several genera and the majority of species, and formed a sister group to the clades B, C, and D with strong statistical support (95% BS, 1.00 PP). Samples of the two new species were placed in the clade A, and one of the new species of *Dacrymycesnaematelioides* formed a non-monophyletic, strongly supported group. The new species *Caloceramultiramosa* was found to be closely related to *C.tibetica* with high supports (85% BS, 1.00 PP), and the two species clustered together with *C.viscosa* with strong supports (91% BS, 1.00 PP). The new species *D.naematelioides* formed a sister lineage to *D.chrysospermus* with 78% bootstrap support and 1.00 posterior probability.

### ﻿Taxonomy

#### ﻿ Campanophyllum microsporum

Taxon classificationFungiAgaricalesCyphellaceae

Y.H. Ma, W.M. Chen & Y.C. Zhao sp. nov.

32E6716A-2BB2-523C-B99A-91A5A6DD606E

853503

Figs 3 , 4 , 5

##### Diagnosis.

*Campanophyllummicrosporum* is characterized by dorsally pseudostipitate pileus, excentric to lateral pseudostipe, crowded lamellae, cylindrical-ellipsoid basidiospores (3.0–4.2 × 1.7–2.2 µm), narrowly clavate to clavate basidia (14.5–23.0 × 3.0–4.0 µm), and cylindrical to clavate cheilocystidia (22.0–55.0 × 5.0–11.0 µm); occurrence in a deciduous forest and solitary, cespitose, scattered, or gregarious habit on rotten wood.

##### Type.

China. Yunnan Province: Jianchuan County, Laojunshan Town (26°35.85'N, 99°40.44'E, elev. 3100 m), on rotten wood, 21 September 2023, Yuan-Hao Ma, Min Zeng & Wei-Min Chen (Holotype: HKAS 133170!, ex-type: YAASM 7187).

##### Etymology.

The epithet “microsporum” refers to the smaller basidiospores compared to *Campanophyllumproboscideum*.

##### Description.

Basidiomata pseudostipitate, dorsally and eccentrically or laterally attached to substrate, occasionally central, pendent, broadly cyphelloid to crepidotoid, lamellate. Pileus 5.0–12.0 × 4.0–9.0 cm, spathulate, flabelliform to rounded-flabelliform, sometimes subcircular; plano-convex when young and applanate when older, margin inrolled, lobate when fully expanded; surface moist, initially pale orange (5A2–4), greyish orange (5B2–3), or light orange (6A2–5), then brownish orange (6C5–6), light brown (6D5–8), often with small stains of darker colors. Context thick, fleshy, whitish, and unchanging in color when injured. Lamellae extending radially from attachment point within pseudostipe, very crowded, sometimes forked, white to off-white, sometimes with small blackish stains. Pseudostipe 0.5–2.5 × 0.4–1.0 cm, concolorous with pileus, discolouring to blackish-ochre (6E5-7, 6F7). Spore print white. Taste mild, odor indistinct.

**Figure 3.**
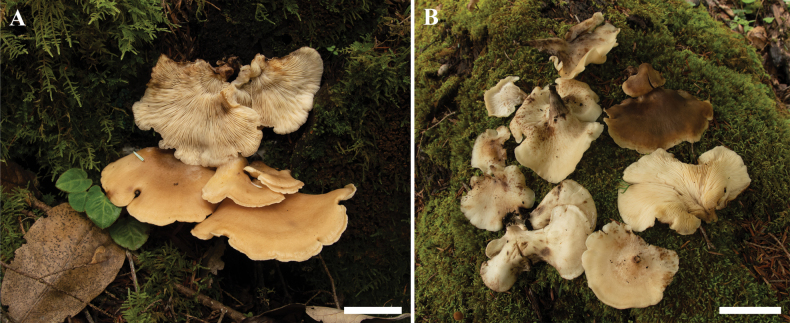
Basidiomata of *Campanophyllummicrosporum* in the field **A**HKAS 133170 (Holotype) **B**HKAS 133169. Photos by Y.H. Ma. Scale bars: 3 cm.

Basidiospores [149/7/4] (2.7–)3.0–4.2(–4.5) × (1.5–)1.7–2.2(–2.6) µm, L_m_ = 3.5 µm, W_m_ = 1.9 µm, Q = 1.4–2.5, Q_m_ = 1.8, cylindrical-ellipsoid, smooth, hyaline, thin-walled, inamyloid. Basidia (13.5–)14.5–23.0(–26.0) × (2.3–)3.0–4.2(–4.6) µm, L_m_ = 17.6 µm, W_m_ = 3.6 µm, Q = 3.6–7.4, Q_m_ = 4.9, narrowly clavate to clavate, 4-spored, sterigma 0.9-2.2 µm. Cheilocystidia abundant, (17.5–)22.0–55.0(–59.0) × (4.2–)5.0–10.8(–13.9) µm, L_m_ = 37.0 µm, W_m_ = 7.2 µm, hyaline, thin-walled, mostly cylindrical to clavate, sometimes lageniform, rod-like, or beaked-utriform, pedunculate (1.9–12.5 × 1.8–4.1 µm). Pleurocystidia not observed. Lamellar trama hyaline, parallel, hyphae 3.6–17.6 µm in diameter, thin- to thick-walled. Pileipellis composed of repent, parallel hyphae, 4.2–11.5 (–17.0) µm in diameter, sometimes with yellow-brown, intracellular pigments. Clamp connections present in all tissues of basidiomata.

**Figure 4.**
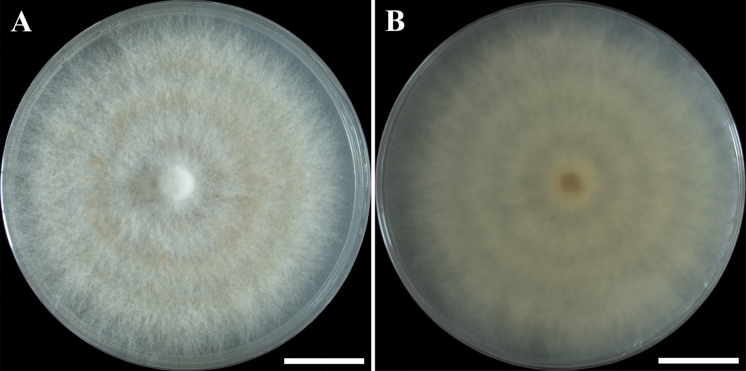
Morphological features of *Campanophyllummicrosporum* on YPD medium after 20 days in the dark in a 9 cm Petri plate (ex-type YAASM 7187) **A** surface of colony **B** reverse of colony. Photos by Y.H. Ma. Scale bars: 2 cm.

Culture characteristics. Colonies grown on YPD reaching 40 mm radius within 20 days at 22 °C in the dark, forming abundant aerial mycelium, usually zonate. Mycelium irregularly cottony, with common clamp connections, pallid mouse gray to pale brown in aerial mycelium with age, easily forming basidiomata in the Petri plate.

**Figure 5.**
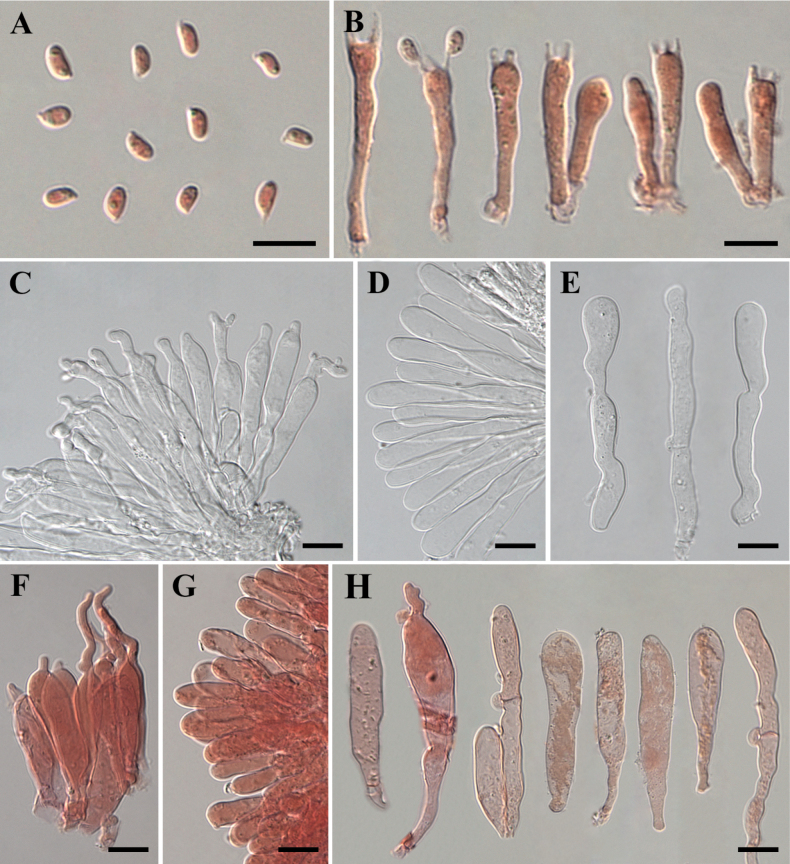
Microscopic structures of *Campanophyllummicrosporum* (Holotype HKAS 133170) **A** basidiospores in Congo red **B** basidia in Congo red **C–H** cheilocystidia (**C–E** in KOH solution **F–H** in Congo red). Photos by Y.H. Ma. Scale bars: 10 µm.

##### Habitat and distribution.

Solitary, cespitose, scattered, or gregarious on rotten wood in a deciduous forest; known from Yunnan, China.

##### Additional specimens examined.

China, Yunnan Province: Jianchuan County, Laojunshan Town, 7 July 2022, Yuan-Hao Ma, Ping Liu & Yong-Chang Zhao (HKAS 133167, HKAS 133168); 26 July 2023, Yuan-Hao Ma & Ping Liu (HKAS 133169).

##### Notes.

*Campanophyllummicrosporum* is similar to *C.proboscideum* in both macro- and micro-morphology, including broadly cyphelloid to crepidotoid basidiomata, spathulate, flabelliform to rounded-flabelliform pileus, and very crowded lamellae; cylindrical-ellipsoid basidiospores, narrowly clavate to clavate basidia. However, several other features can distinguish the two species. Morphologically, the new species have smaller basidiospores (3.0–4.2 × 1.7–2.2 µm vs. 4–4.5 × 2–3 µm), slenderer and longer basidia (14.5–23.0 × 3.0–4.2 µm vs. 14–17 × 4.5–5.0 µm), and larger cheilocystidia (22.0–55.0 × 5.0–10.8 µm vs. 18–25 × 9–11 µm) (Cifuentes et al. 2003).

#### ﻿ Calocera multiramosa

Taxon classificationFungiDacrymycetalesDacrymycetaceae

Y.H. Ma, W.M. Chen & Y.C. Zhao sp. nov.

9A58478F-8AA2-51C5-8359-DBB42C73948E

853504

Figs 6 , 7 , 8

##### Diagnosis.

*Caloceramultiramosa* differs from other species of the genus by yellowish to orange basidiomata, dendroid and dichotomously branches, branched, smooth, thin-walled marginal hyphae (2.0–4.8 µm), branched, thin-walled internal hyphae (2.9–10.0 µm), cylindrical to clavate basidia (36.5–52.5 × 4.0–6.0 µm), 1–5-septate, navicular or reniform basidiospores (10.4–16.7 × 5.2–7.4 µm), occurrence in a deciduous or coniferous forest, occasionally scattered habit on standing timber.

##### Type.

China. Yunnan Province: Shangri-La County, Pudacuo National Park (27°50.61'N, 99°57.03'E, elev. 3800 m), on standing timber, 17 August 2020, Yuan-Hao Ma, Hong-Mei Chai & Wei-Min Chen (Holotype: HKAS 133171!).

##### Etymology.

The epithet “multiramosa” refers to abundant branches of basidiomata.

##### Description.

Basidiomata stipitate, fasciculate, usually geminate, occasionally scattered, gelatinous, 1.5–4.0 cm in height, tough, dendroid and dichotomously branched, cylindrical or flattened, surface smooth, yellowish to orange (5B8, 6A8, 6B7–8), 0.3–0.5 cm in diameter at the upper branching part. Marginal hyphae on sterile surfaces cylindrical, branched, smooth, straight or flexuous, septate, thin-walled, hyaline, 2.0–4.8 µm in diameter. Internal hyphae branched, septate, thin-walled, hyaline, 2.9–10.0 µm in diameter. Hymenium limited to the upper surface of basidomata, amphigenous, composed of basidia and simple cylindrical hyphidia; hyphidia hyaline or pale yellow, smooth, thin-walled. Subhymenial hyphae hyaline, smooth or scabrous, thin- or slightly thick-walled, 2.5–7.3 µm in diameter. Basidia cylindrical to clavate, hyaline or pale yellow, thin-walled, becoming bifurcate when mature, (33.5–)36.5–52.5(–55.0) × (3.5–)3.8–6.1(–6.4) µm, L_m_ = 45.1 µm, W_m_ = 4.9 µm, sometimes with many septa. Basidiospores [102/3/3], navicular or reniform, straight or curved, with a small apiculum at the top, thin-walled with thin septa, hyaline to pale yellow, sometimes with oil drops when young and in the germination stage, (6.5–)10.4–16.7(–17.0) × (4.5–)5.2–7.4(–8.8) µm, L_m_ = 14.3 µm, W_m_ = 6.3 µm, Q = (1.4–)1.6–2.7(–2.8), Q_m_ = 2.3, 1–5-septate at maturity. Germination with conidia by abnormally developing basidia with lots of septa, by hyphae with septa, or by germ tubes. Clamp connections absent in all tissues of the basidiomata.

**Figure 6.**
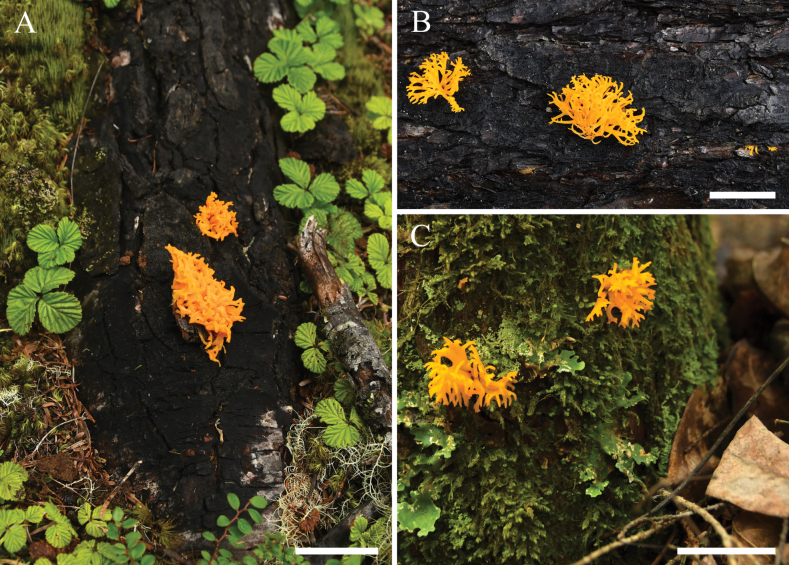
Basidiomata of *Caloceramultiramosa***A**HKAS 133171 (Holotype) **B**HKAS 133172 **C**HKAS 133173. Photos by Y.H. Ma. Scale bars: 3 cm.

##### Habitat and distribution.

Geminate, occasionally scattered on standing timber in a deciduous or coniferous forest; known from Yunnan, China.

**Figure 7.**
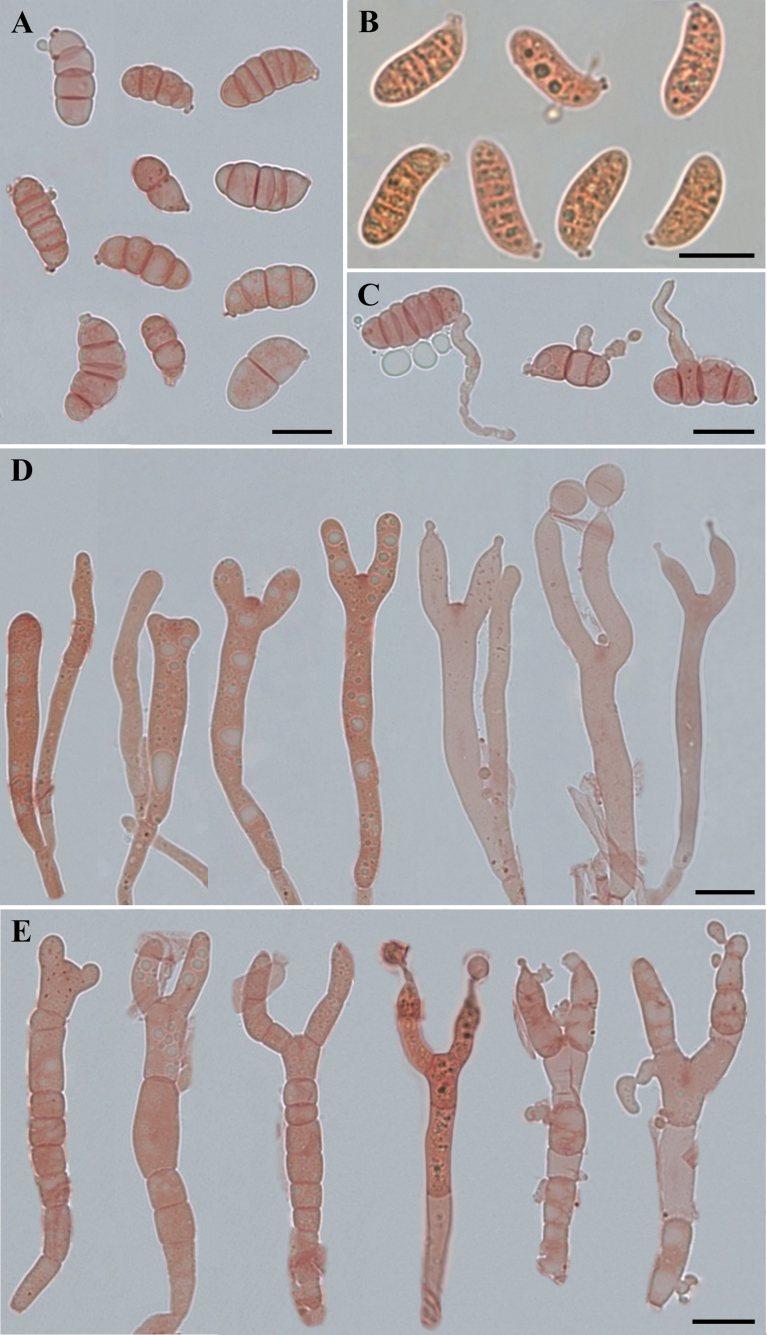
Microscopic structures of *Caloceramultiramosa* in Congo red (Holotype HKAS 133171) **A**, **B** basidiospores **C** germinating basidiospores **D** probasidia, developing basidia and hyphidia **E** abnormal developing basidia with septa and geminations. Photos by Y.H. Ma. Scale bars: 10 µm.

##### Additional specimens examined.

China. Yunnan Province: Shangri-La County, Pudacuo National Park, 28 August 2021, Yuan-Hao Ma, Ping Liu & Yong-Chang Zhao (HKAS 133172); Jianchuan County, Laojunshan Town, 26 July 2023, Yuan-Hao Ma & Ping Liu (HKAS 133173).

**Figure 8.**
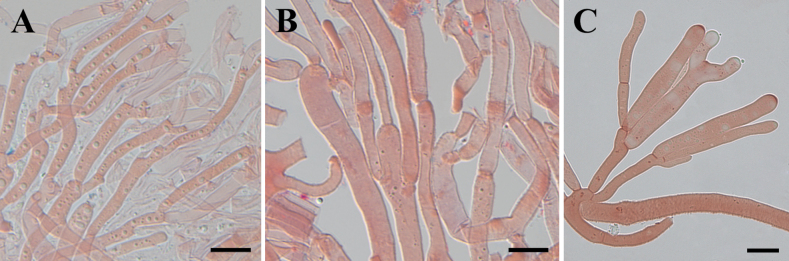
Microscopic structures of *Caloceramultiramosa* in Congo red (Holotype HKAS 133171) **A** marginal hyphae **B** internal hyphae **C** subhymenial hyphae, probasidia, developing basidia, and hyphidia. Photos by Y.H. Ma. Scale bars: 10 µm.

##### Notes.

*Caloceramultiramosa* resembles *C.tibetica*, *C.viscosa* and *C.mangshanensis* in dendrite basidiomata. However, *C.multiramosa* is distinguished from *C.tibetica* by larger basidiospores (10.4–16.7 × 5.2–7.4 µm vs. 9.0–13.0 × 5.0–6.0 µm) with different septa (1–5 vs. 3–4) (Fan et al. 2021); *C.multiramosa* differs from *C.viscosa* in larger basidia (36.5–52.5 × 3.8–6.1 µm vs. 23–42 × 3–4.5 µm) and basidiospores with different septa (1–5 vs. 0–1) (McNabb 1965; Shirouzu et al. 2009). *C.multiramosa* can be distinguished from *C.mangshanensis* by larger (10.4–16.7 × 5.2–7.4 µm vs. 10.0–13.0 × 4.5–5.5 µm), more septate (1–5 vs. 0–1) basidiospores (Liu and Fan 1989). The new species grows on angiosperm and gymnosperm wood, while *C.tibetica* and *C.viscosa* only grows on gymnosperm wood and *C.mangshanensis* only grows on decayed angiosperm wood (McNabb 1965; Liu and Fan 1989; Oberwinkler 2014). *C.multiramosa* can be distinguished from *C.cornea* by the size of the basidiomata (1.5–4.0 cm vs. 0.1–0.5 cm high) (Shirouzu et al. 2009), and *C.furcata* by the mature basidiospores with different septa (1–5 vs. 1–3) (McNabb 1965). The specimen of *C.multiramosa*, collected from the Laojun Mountain could not be designated as the holotype because of many immature basidiospores. Therefore, the specimen of *C.multiramosa* collected from a coniferous forest in the Pudacuo National Park was designated as the holotype.

#### ﻿ Dacrymyces naematelioides

Taxon classificationFungiDacrymycetalesDacrymycetaceae

Y.H. Ma, W.M. Chen & Y.C. Zhao sp. nov.

BF63772A-2D5D-55A6-AA67-41FDC5F695C3

853505

Figs 9 , 10 , 11

##### Diagnosis.

*Dacrymycesnaematelioides* differs from other species of the genus by stipitate and cerebriform basidiomata, smooth or roughened, simple or branched, septate marginal hyphae (3.0–8.5 µm), smooth or roughened, thin-walled, branched, and septate internal hyphae (2.3–11.0 µm), cylindrical to clavate, smooth or roughened basidia (38.5–79.5 × 6.5–10.6 µm), broadly elliptic-fusiform, 7-septate mature basidiospores (18.5–28.6 × 8.9–13.8 µm), the absence of clamp connections, occurrence in a deciduous forest, and fasciculate, gregarious, or scattered habit on rotten wood.

**Figure 9.**
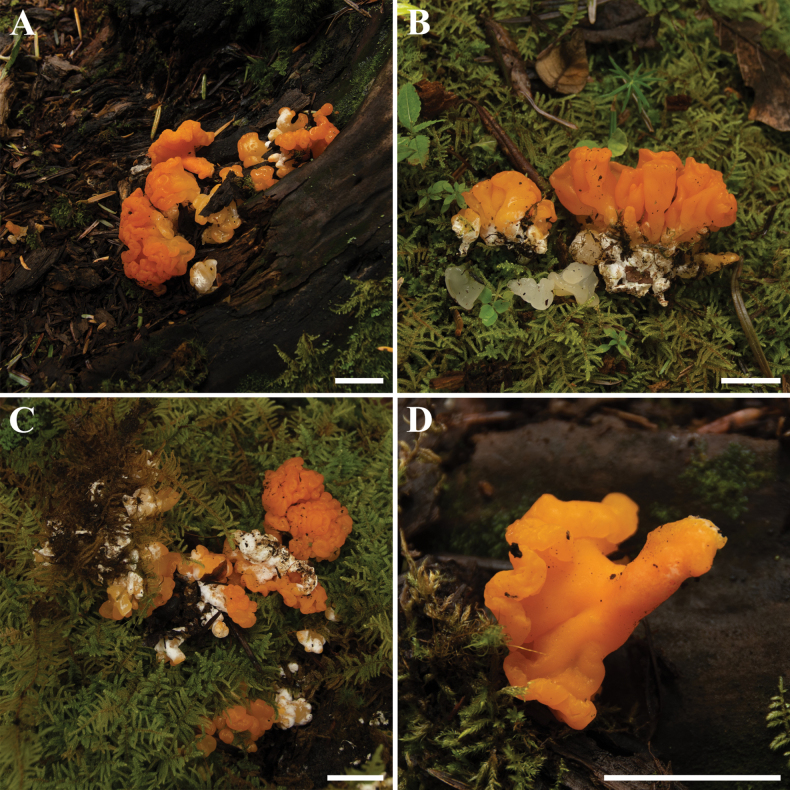
Basidiomata of *Dacrymycesnaematelioides***A–D**HKAS 133174 (Holotype). Photos by Y.H. Ma. Scale bars: 3 cm.

##### Type.

China. Yunnan Province: Jianchuan County, Laojunshan Town (26°35.86'N, 99°40.46'E, elev. 3100 m), 21 September 2023, Yuan-Hao Ma, Min Zeng & Wei-min Chen (Holotype: HKAS 133174!).

**Figure 10.**
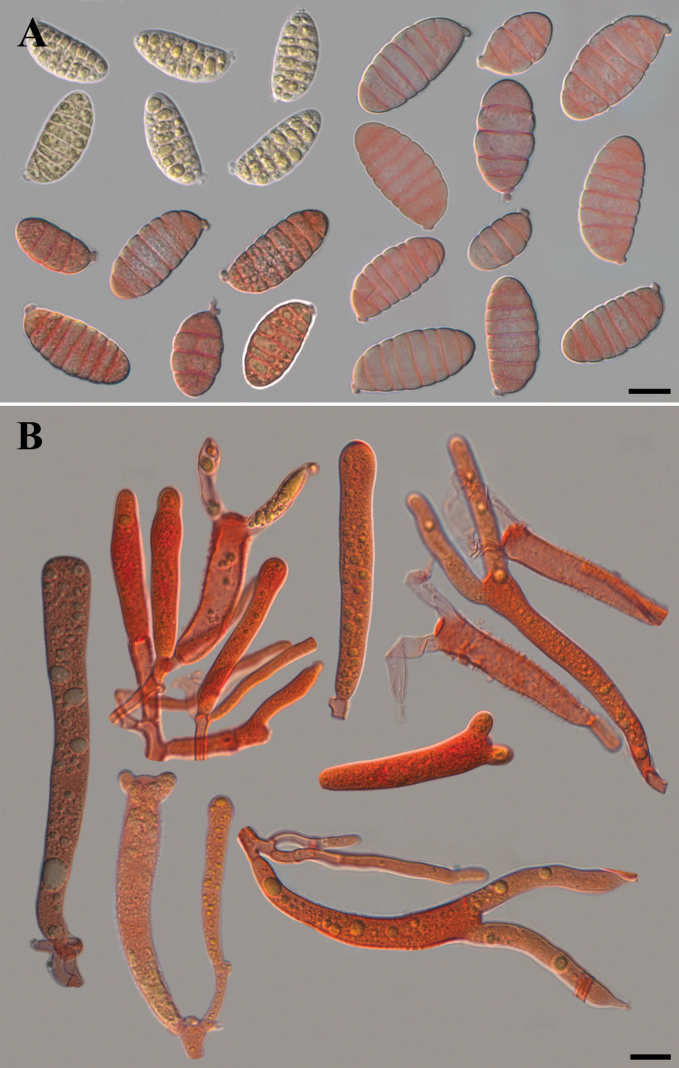
Microscopic structures of *Dacrymycesnaematelioides* (Holotype HKAS 133174) **A** immature and mature basidiospores in Congo red and KOH solution **B** probasidia, developing basidia and hyphidia in Congo red. Photos by Y.H. Ma. Scale bars: 10 µm.

##### Etymology.

The epithet “naematelioides” refers to the similarity of the new species in terms of macromorphological features to *Naemateliaaurantialba*.

##### Description.

Basidiomata stipitate, fasciculate and conspicuous, gregarious or scattered, gelatinous when fresh, cerebriform, 2.5–4.5 cm high, surface smooth, orange to light brown (6A8, 6D7–8), occasionally colorless, stipe flat cylindrical, usually with white hairs. Marginal hyphae on sterile surfaces of basidiocarps cylindrical, simple or branched, smooth or roughened, straight or flexuous, septate, thick-walled, hyaline, 3.0–8.5 µm in diameter. Internal hyphae branched, septate, thin-walled, hyaline, smooth or roughened, 2.3–11.0 µm in diameter. Hymenium limited to the upper surface of the basidoma, amphigenous, composed of basidia and simple cylindrical hyphidia; hyphidia hyaline or pale yellow, smooth, thin-walled. Subhymenial hyphae, smooth or roughened, thin- to thick-walled, 2.5–5.3 µm in diameter. Basidia cylindrical to clavate, smooth or roughened, pale yellow, thin-walled, becoming bifurcate, (30.0–)38.5–79.5(–83.5) × (5.5–)6.5–10.6(–11.1) µm, L_m_ = 60.2 µm, W_m_ = 8.3 µm. Basidiospores [95/5/1], broadly and elliptic-fusiform, with a small apiculum at the base, thin-walled, pale yellow, with oil drops when young, (16.5–)18.5–28.5(–29.5) × (8.7–)8.9–13.8(–14.6) µm, L_m_ = 23.9 µm, W_m_ = 11.0 µm, Q = (1.5–)1.8–2.4(–2.5), Q_m_ = 2.2, usually 7–septate, rarely 3– or 4– septate at maturity. Germination not observed. Clamp connections absent in all tissues of the basidiomata.

**Figure 11.**
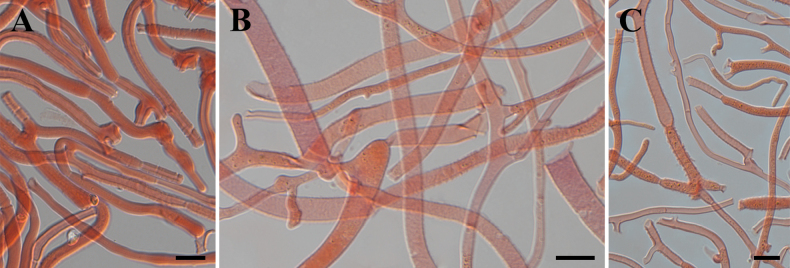
Microscopic structures of *Dacrymycesnaematelioides* (Holotype HKAS 133174) **A** marginal hyphae **B** internal hyphae **C** subhymenial hyphae. Photos by Y.H. Ma. Scale bars: 10 µm.

##### Habitat and distribution.

Fasciculate, gregarious, or scattered habit on rotten wood, and occurrence in a deciduous forest; known from Yunnan, China.

##### Notes.

*Dacrymycesnaematelioides* resembles *D.chrysospermus* and *D.dictyosporus* in shape and size of basidiomata. Microscopically, *D.chrysospermus* differs from *D.naematelioides* by narrower basidia (4–6.5 µm vs. 6.5–10.6 µm in width) and smaller basidiospores (16.5–23 × 5–7.5 µm vs. 18.5–28.6 ×8.9–13.8 µm), and *D.dictyosporus* differs by smooth basidia and thick-walled basidiospores (Martin et al. 1958; McNabb 1973).

## ﻿Discussion

In this study, we described three new species from Yunnan Province, China, based on morphological evidence and multi-locus phylogenic analyses. The identified morphological features of *Campanophyllum* include dorsally pseudostipitate pileus, excentric to lateral pseudostipe, and crowded lamellae (Cifuentes et al. 2003; Reschke et al. 2021). The specimen of *Campanophyllum* sp. (Voucher NEHU.MBSRJ. 38) reported from India formed a sister lineage to *C.microsporum* with strong supports (100% BS, 1.00 PP). The specimen (Voucher NEHU.MBSRJ. 38) is presumably a new species in the genus *Campanophyllum* based on the phylogenetic trees (Fig. 1), but it needs to be further confirmed. Meanwhile, our research indicates that more new species of this genus will be discovered in China. However, their habitat is in decline and disappearing.

The species of *Calocera* in the family Dacrymycetaceae are typically distinguished morphologically based on simple or forked clavarioid basidiocarps (Oberwinkler 2014), but the genus *Dacrymyces* includes more than 30 species with variable basidiomata including pulvinate, discoid, turbinate, spathulate, flabellate, and cylindrical forms (McNabb 1973; Shirouzu et al. 2009). Several species of *Dacrymyces* are morphologically close to *Calocera* by sharing spathulate or cylindrical basidioma, and yet they can be distinguished by some other morphological features, such as the septa of the basidiospores, morphology of the basidia, and morphology of the marginal hyphae. More appropriate genus boundaries and definitions can be obtained by studying detailed morphological and molecular data on more specimens of Dacrymycetaceae. Continuing collection efforts and herbarium searches in unidentified Dacrymycetaceae will certainly uncover more new species (Savchenko et al. 2021).

Phylogenetic analyses, based on two combined loci (ITS, LSU), as well as morphological characteristics, are important for the identification of *Calocera* and *Dacrymyces* species. The two newly proposed species formed separate branches on the phylogenetic trees with strong statistical support, and the phylogenies for the genera presented here were found to be similar to those of previous studies (Shirouzu et al. 2013). The results of our study indicated that the specimens of *Dacrymycesnaematelioides* collected from the same locality formed two distinct clades in the phylogenetic analysis (Fig. 2) with strong statistical support (100% BS, 1.00 PP), but there is no marked difference in their morphological characteristics. This suggests that there may be some variation in this species at the molecular level.

The abnormal developing basidia and probasidia with a lot of septa in specimens of *Caloceramultiramosa* were also observed clearly under the microscope, and sometimes they can germinate with microconidia in the basidiomata. This microscopic feature may also be useful in identifying species in the genus *Calocera*. The surface of the basidia of the *Dacrymycesnaematelioides* is smooth or roughened (Fig. 9). However, the surface features of the basidia did not seem to have been noted in much of the literature, and it is likely that in most species the surface of the basidia is smooth or has only one morphological feature.

## Supplementary Material

XML Treatment for Campanophyllum microsporum

XML Treatment for Calocera multiramosa

XML Treatment for Dacrymyces naematelioides

## ﻿Additional information

### Author ORCIDs

Yuan-Hao Ma https://orcid.org/0000-0001-6638-8931

Ping Liu https://orcid.org/0000-0002-1345-9887

Hong-Mei Chai https://orcid.org/0000-0003-4893-4315

Yi-Yun Guo https://orcid.org/0009-0005-3326-9588

Wei-Min Chen https://orcid.org/0000-0001-5595-1514

Yong-Chang Zhao https://orcid.org/0000-0003-1494-4259

### Data availability

All of the data that support the findings of this study are available in the main text.

## References

[B1] Ahmed SA de Hoog S Kim J Crozier J Thomas SE Stielow B Stevens DA (2020) *Gloeostereumcimri*, a novel shelf fungus isolated from a human pulmonary cyst. Emerging Microbes & Infections 9(1): 1114–1122. 10.1080/22221751.2020.1769499 32475225 PMC8284975

[B2] Binder M Hibbett DS Larsson KH Larsson E Langer E Langer G (2005) The phylogenetic distribution of resupinate forms across the major clades of mushroom‐forming fungi (Homobasidiomycetes). Systematics and Biodiversity 3(2): 113–157. 10.1017/S1477200005001623

[B3] Borthakur M Gurung AB Bhattacharjee A Joshi SR (2020) Analysis of the bioactive metabolites of the endangered mexican lost fungi *Campanophyllum* – a report from India. Mycobiology 48(1): 58–69. 10.1080/12298093.2020.1723388 32158607 PMC7048233

[B4] Castro-Santiuste S Sierra S Guzman-Davalos L Cifuentes J Evans T Martinez-Gonzalez CR Alvarado-Sizzo H Luna-Vega I (2020) *Dacryopinax* (Fungi: Dacrymycetales) in Mexico. Phytotaxa 446(1): 6–22. 10.11646/phytotaxa.446.1.2

[B5] Cifuentes J Petersen RH Hughes K (2003) *Campanophyllum*: A new genus for an old species name. Mycological Progress 2(4): 285–295. 10.1007/s11557-006-0066-z

[B6] Darriba D Taboada GL Doallo R Posada D (2012) jModelTest 2: More models, new heuristics and parallel computing. Nature Methods 9(8): 772. 10.1038/nmeth.2109 PMC4594756 22847109

[B7] Fan L-F Wu Y-D Wu F Dai Y-C (2021) *Caloceratibetica* sp. nov. (Dacrymycetaceae, Dacrymycetales) from southwestern China. Phytotaxa 500(2): 133–141. 10.11646/phytotaxa.500.2.6

[B8] Guindon S Gascuel O (2003) A simple, fast, and accurate algorithm to estimate large phylogenies by maximum likelihood. Systematic Biology 52(5): 696–704. 10.1080/10635150390235520 14530136

[B9] Hall TA (1999) BioEdit: A user-friendly biological sequence alignment editor and analysis program for Windows 95/98/NT. Nucleic Acids Symposium Series 41: 95–98.

[B10] Hibbett DS Binder M (2002) Evolution of complex fruiting-body morphologies in homobasidiomycetes. Proceedings. Biological Sciences 269(1504): 1963–1969. 10.1098/rspb.2002.2123 12396494 PMC1691125

[B11] Holec J Kunca V Kříž M Zehnálek P (2022) *Cyphelladigitalis* (Fungi, Agaricales) – new data on ITS barcode, ecology and distribution in the Czech Republic and Slovakia. Czech Mycology 74(1): 77–92. 10.33585/cmy.74106

[B12] Jang Y Jang S Min M Hong JH Lee H Lee H Lim YW Kim JJ (2015) Comparison of the diversity of Basidiomycetes from dead wood of the Manchurian fir (*Abiesholophylla*) as evaluated by fruiting body collection, mycelial isolation, and 454 sequencing. Microbial Ecology 70(3): 634–645. 10.1007/s00248-015-0616-5 25933635

[B13] Katoh K Standley DM (2013) MAFFT multiple sequence alignment software version 7: Improvements in performance and usability. Molecular Biology and Evolution 30(4): 772–780. 10.1093/molbev/mst010 23329690 PMC3603318

[B14] Kornerup A Wanscher JH (1978) Methuen Handbook of Colour. 3^rd^ edn. Eyre Methuen, London, 252 pp.

[B15] Kozlov AM Darriba D Flouri T Morel B Stamatakis A (2019) RAxML-NG: A fast, scalable and user-friendly tool for maximum likelihood phylogenetic inference. Bioinformatics 35(21): 4453–4455. 10.1093/bioinformatics/btz305 31070718 PMC6821337

[B16] Larsson KH (2007) Molecular phylogeny of *Hyphoderma* and the reinstatement of *Peniophorella*. Mycological Research 111(2): 186–195. 10.1016/j.mycres.2006.10.002 17164083

[B17] Lian YP Tohtirjap A Wu F (2022) Two New Species of *Dacrymyces* (Dacrymycetales, Basidiomycota) from Southwestern China. Diversity 14(5): 379. 10.3390/d14050379

[B18] Liu B Fan L (1989) Two new species of Dacrymycetaceae from China. Acta Microbiologica Sinica 8(1): 22–24.

[B19] Liu B Fan L (1990) New species and new variety of Dacrymycetaceae in China. Acta Microbiologica Sinica 9(1): 12–19.

[B20] Liu B Fan L Tao K (1988) Five new species of Dacrymycetaceae from China. Acta Mycologica Sinica 7(1): 1–6.

[B21] Martin GW Luttrell ES Karling JS Stuehling Jr JJ Ziegler AW (1958) Notes and brief articles. Mycologia 50(6): 939–948. 10.1080/00275514.1958.12024785

[B22] Matheny PB Curtis JM Hofstetter V Aime MC Moncalvo JM Ge ZW Yang ZL Slot JC Ammirati JF Baroni TJ Bougher NL Hughes KW Lodge DJ Kerrigan RW Seidl MT Aanen DK DeNitis M Daniele GM Desjardin DE Kropp BR Norvell LL Parker A Vellinga EC Vilgalys R Hibbett DS (2006) Major clades of Agaricales: A multilocus phylogenetic overview. Mycologia 98(6): 982–995. 10.1080/15572536.2006.11832627 17486974

[B23] McNabb RFR (1965) Taxonomic studies in the Dacrymycetaceae: II. *Calocera* (Fries) Fries. New Zealand Journal of Botany 3(1): 31–58. 10.1080/0028825X.1965.10428712

[B24] McNabb RFR (1973) Taxonomic studies in the dacrymycetaceae VIII. *Dacrymyces* Nees ex Fries. New Zealand Journal of Botany 11(3): 461–524. 10.1080/0028825X.1973.10430296

[B25] Merlet L Wiseman MS Serdani M Putnam ML (2018) First Report of Silver Leaf Caused by *Chondrostereumpurpureum* on *Vacciniumcorymbosum* in Oregon. Plant Disease 102(10): 2041–2041. 10.1094/PDIS-02-18-0312-PDN

[B26] Mittermeier RA Turner WR Larsen FW Brooks TM Gascon C (2011) Global Biodiversity Conservation: The Critical Role of Hotspots. In: Zachos FE Habel JC (Eds) Biodiversity Hotspots: Distribution and Protection of Conservation Priority Areas.Springer, Berlin, Heidelberg, 3–22. 10.1007/978-3-642-20992-5_1

[B27] Moncalvo JM Lutzoni FM Rehner SA Johnson J Vilgalys R (2000) Phylogenetic relationships of agaric fungi based on nuclear large subunit ribosomal DNA sequences. Systematic Biology 49(2): 278–305. 10.1093/sysbio/49.2.278 12118409

[B28] Na Q Liu ZW Zeng H Ke BR Song ZZ Cheng XH Ge YP (2022) Taxonomic studies of bluish *Mycena* (Mycenaceae, Agaricales) with two new species from northern China. MycoKeys 90: 119–145. 10.3897/mycokeys.90.78880 36760418 PMC9849084

[B29] Nees von Esenbeck, CGD (1816–7) Das System der Pilze und Schwämme: ein Versuch. Stahelschen Buchhandlung, Wurzburg, 329 pp. 10.5962/bhl.title.110007

[B30] Oberwinkler F (2014) Dacrymycetes. In: McLaughlin DJ Spatafora JW (Eds) Systematics and Evolution: Part A, The Mycota.Springer, Berlin, Heidelberg, 357–372. 10.1007/978-3-642-55318-9_13

[B31] Parmasto E Hallenberg N (2000) A taxonomic study of phlebioid fungi (Basidiomycota). Nordic Journal of Botany 20(1): 105–118. 10.1111/j.1756-1051.2000.tb00740.x

[B32] Reschke K Lotz-Winter H Fischer CW Hofmann TA Piepenbring M (2021) New and interesting species of Agaricomycetes from Panama. Phytotaxa 529(1): 1–26. 10.11646/phytotaxa.529.1.1

[B33] Robles CA Lopez SE McCargo PD Carmarán CC (2015) Relationships between fungal endophytes and wood-rot fungi in wood of *Platanusacerifolia* in urban environments. Canadian Journal of Forest Research 45(7): 929–936. 10.1139/cjfr-2014-0560

[B34] Ronquist F Teslenko M van der Mark P Ayres DL Darling A Höhna S Larget B Liu L Suchard MA Huelsenbeck JP (2012) MrBayes 3.2: Efficient bayesian phylogenetic inference and model choice across a large model space. Systematic Biology 61(3): 539–542. 10.1093/sysbio/sys029 22357727 PMC3329765

[B35] Savchenko A Zamora JC Shirouzu T Spirin V Malysheva V Kõljalg U Miettinen O (2021) Revision of *Cerinomyces* (Dacrymycetes, Basidiomycota) with notes on morphologically and historically related taxa. Studies in Mycology 99(1): 100117. 10.1016/j.simyco.2021.100117 34934464 PMC8645972

[B36] Shirouzu T Hirose D Tokumasu S (2007) Sequence analyses of the 28S rRNA gene D1/D2 region suggest *Dacrymyces* (Heterobasidiomycetes, Dacrymycetales) is polyphyletic. Mycoscience 48(6): 388–394. 10.1007/S10267-007-0378-0

[B37] Shirouzu T Hirose D Tokumasu S (2009) Taxonomic study of the Japanese Dacrymycetes. Persoonia - Molecular Phylogeny and Evolution of Fungi 23: 16–34. 10.3767/003158509X468443 PMC2802733 20198158

[B38] Shirouzu T Hirose D Oberwinkler F Shimomura N Maekawa N Tokumasu S (2013) Combined molecular and morphological data for improving phylogenetic hypothesis in Dacrymycetes. Mycologia 105(5): 1110–1125. 10.3852/12-147 22962355

[B39] Shirouzu T Uno K Hosaka K Hosoya T (2016) Early-diverging wood-decaying fungi detected using three complementary sampling methods. Molecular Phylogenetics and Evolution 98: 11–20. 10.1016/j.ympev.2016.01.015 26850687

[B40] Shirouzu T Hosaka K Nam KO Weir BS Johnston PR Hosoya T (2017) Phylogenetic relationships of eight new Dacrymycetes collected from New Zealand. Persoonia - Molecular Phylogeny and Evolution of Fungi 38: 156–169. 10.3767/003158517X695280 PMC5645182 29151631

[B41] Vaidya G Lohman DJ Meier R (2011) SequenceMatrix: Concatenation software for the fast assembly of multi-gene datasets with character set and codon information. Cladistics 27(2): 171–180. 10.1111/j.1096-0031.2010.00329.x 34875773

[B42] Varga T Krizsán K Földi C Dima B Sánchez-García M Sánchez-Ramírez S Szöllősi GJ Szarkándi JG Papp V Albert L Andreopoulos W Angelini C Antonín V Barry KW Bougher NL Buchanan P Buyck B Bense V Catcheside P Chovatia M Cooper J Dämon W Desjardin D Finy P Geml J Haridas S Hughes K Justo A Karasiński D Kautmanova I Kiss B Kocsubé S Kotiranta H LaButti KM Lechner BE Liimatainen K Lipzen A Lukács Z Mihaltcheva S Morgado LN Niskanen T Noordeloos ME Ohm RA Ortiz-Santana B Ovrebo C Rácz N Riley R Savchenko A Shiryaev A Soop K Spirin V Szebenyi C Tomšovský M Tulloss RE Uehling J Grigoriev IV Vágvölgyi C Papp T Martin FM Miettinen O Hibbett DS Nagy LG (2019) Megaphylogeny resolves global patterns of mushroom evolution. Nature Ecology & Evolution 3(4): 668–678. 10.1038/s41559-019-0834-1 30886374 PMC6443077

[B43] Vilgalys R Hester M (1990) Rapid genetic identification and mapping of enzymatically amplified ribosomal DNA from several *Cryptococcus* species. Journal of Bacteriology 172(8): 4238–4246. 10.1128/jb.172.8.4238-4246.1990 2376561 PMC213247

[B44] Vizzini A Consiglio G Marchetti M Borovička J Campo E Cooper J Lebeuf R Ševčíková H (2022) New data in Porotheleaceae and Cyphellaceae: Epitypification of *Prunulusscabripes* Murrill, the status of *Mycopan* Redhead, Moncalvo & Vilgalys and a new combination in *Pleurella* Horak emend. Mycological Progress 21(4): 44. 10.1007/s11557-022-01795-z

[B45] Vu D Groenewald M de Vries M Gehrmann T Stielow B Eberhardt U Al-Hatmi A Groenewald JZ Cardinali G Houbraken J Boekhout T Crous PW Robert V Verkley GJM (2019) Large-scale generation and analysis of filamentous fungal DNA barcodes boosts coverage for kingdom fungi and reveals thresholds for fungal species and higher taxon delimitation. Studies in Mycology 92(1): 135–154. 10.1016/j.simyco.2018.05.001 29955203 PMC6020082

[B46] Wei RX Ge YP Qi LL Han MH Zeng H Hu YP Zou L Cheng XH Wu XM Na Q (2024) Revealing brownish *Mycena* diversity in China: New discoveries and taxonomic insights. Journal of Fungi 10(6): 439. 10.3390/jof10060439 38921425 PMC11204746

[B47] White TJ Bruns T Lee S Taylor J (1990) Amplification and direct sequencing of fungal ribosomal RNA genes for phylogenetics. In: Innis MA Gelfand DH Sninsky JJ White TJ (Eds) PCR Protocols: a Guide to Methods and Applications.Academic Press, San Diego, 315–322. 10.1016/B978-0-12-372180-8.50042-1

[B48] Wu CY Zhu YC (1987) Vegetation in Yunnan. Science Press, Beijing.

[B49] Zamora JC Ekman S (2020) Phylogeny and character evolution in the Dacrymycetes, and systematics of Unilacrymaceae and Dacryonaemataceae fam. nov. Persoonia - Molecular Phylogeny and Evolution of Fungi 44: 161–205. 10.3767/persoonia.2020.44.07 PMC7567964 33116340

[B50] Zamora JC Savchenko A González-Cruz Á Prieto-García F Olariaga I Ekman S (2022) *Dendrodacrys*: A new genus for species with branched hyphidia in *Dacrymyces* s.l., with the description of four new species. Fungal Systematics and Evolution 9(1): 27–42. 10.3114/fuse.2022.09.04 35978985 PMC9355100

[B51] Zhang Y Zhou DQ Zhao Q Zhou TX Hyde KD (2010) Diversity and ecological distribution of macrofungi in the Laojun Mountain region, southwestern China. Biodiversity and Conservation 19(12): 3545–3563. 10.1007/s10531-010-9915-9

